# An African perspective on the Water-Energy-Food nexus

**DOI:** 10.1038/s41598-023-43606-9

**Published:** 2023-10-06

**Authors:** Gareth B. Simpson, Graham P. W. Jewitt, Tafadzwanashe Mabhaudhi, Cuthbert Taguta, Jessica Badenhorst

**Affiliations:** 1 WSP, Newcastle, Australia; 2 IHE Delft Institute for Water Education, Delft, The Netherlands; 3 Civil Engineering and Geosciences, Delft University of Technology, Delft, The Netherlands; 4 Centre for Water Resources Research, College of Agriculture, Engineering and Science, University of KwaZulu-Natal, Pietermaritzburg, South Africa; 5 Centre for Transformative Agricultural and Food Systems, College of Agriculture, Engineering, and Science, University of KwaZulu-Natal, Pietermaritzburg, South Africa; 6 International Water Management Institute, West Africa Office, Accra, Ghana; 7 School of Engineering, College of Agriculture, Engineering and Science, University of KwaZulu-Natal, P. Bag X01, Pietermaritzburg, 3209 South Africa; 8 Worley, London, England

**Keywords:** Environmental impact, Civil engineering

## Abstract

There is a need to address resource security and distributional justice in developing countries. People need water, energy, and food to sustain their livelihoods, grow economies, and achieve sustainable development. The interactions between these resource sectors form the crux of water-energy-food (WEF) nexus assessments. In this study, we have utilised the WEF Nexus Index to analyse the WEF nexus of 54 African nations. The results from the analysis were used to illustrate the opportunities and constraints for future development. Generally, African countries are performing sub-optimally in the WEF Nexus Index due to the insecurity of water, energy and/or food. The performance of countries varies with context, highlighting the need for contextual analysis in identifying challenges and potential solutions. Implementation of interventions for achieving WEF security needs to be planned from an integrated perspective to optimise synergies and minimize trade-offs. Implementation of the WEF nexus approach towards simultaneous security of WEF resources has potential to improve the WEF nexus. For example and for many African countries, policies that undergird investments in energy supply projects are needed to unlock available freshwater resources and meet food requirements—energy is shown to be a critical enabler of development. Such projects can be utilised to enhance the ability of farmers to manage water through drought-proofing rainfed agriculture, an increase in irrigation development, or both. WEF nexus-based studies, policies, and projects must be focused on the direct and indirect achievement of SDGs 1, 2, 6, 7, and 13, both in terms of access and availability, to ensure distributional justice, especially in the African context. Such actions, combined with broad public participation, can have a ripple effect on other SDGs such as SDGs 5, 10, and 17, thereby reducing inequalities and building partnerships to attain these aspirational goals. The assessment of Africa’s relatively low scores in terms of the WEF Nexus Index does not represent a negative narrative. Instead, it provides an entry point to identifying hotspots and understanding the underlying challenges, through which more detailed analyses can lead to identified solutions and policies. Many African countries are trapped in an environment that could be termed a ‘poverty-unemployment-inequality nexus’ (due to the interlinkages that exist between these ‘wicked’ problems). The WEF Nexus Index provides high-level insights into these opportunities.

## Introduction

Rapid global population growth has resulted in an increased demand for many resources^1,2^, including minerals, building materials, water, energy, and food^3–5^. With the anthropogenic effects of development showing no signs of abating^6,7^ despite the global impacts of the COVID-19 pandemic and the increasing consumption of a burgeoning middle class (especially in Africa and Asia), increasing pressure is being placed on the planet’s limited resources^8^. It has been projected that worldwide energy demand will effectively double by 2050, while water and food demand will rise by over 50%^9–12^. The war in Ukraine has resulted in additional human and resource crises, amongst them, global turmoil regarding energy (e.g., fuel, gas), food (e.g., wheat, maize, barley, vegetable oil) and fertilizer prices, supply, and demand^13–15^.

Persistent challenges that complicate achieving the simultaneous security and sustainability of water, energy, and food resources are motivating a shift from traditional sectoral thinking towards integrated approaches to resource management, for example the nexus planning approach. Derived from the term “nexus” which represents the interactions between two or more elements, the water-energy-food (WEF) nexus approach seeks to study and understand the inextricable connections between these three resource sectors, and to manage the synergies, conflicts, and trade-offs while ensuring the integrity of land, the environment and ecosystems^16–18^. The WEF nexus is recognised as a means of facilitating progress towards the relevant sector-related Sustainable Development Goals (SDGs), i.e. SDGs 2, 6, and 7^18^. This is evidenced by several policy briefs and collaborative research initiatives between the global North and South^19,20^. However, there is little empirical evidence to guide and motivate implementation, and few practical tools available to support the implementation and assess the performance of such initiatives. Further, there is a resounding call to migrate from ‘nexus thinking’ to ‘nexus action’^18,21,22^. This paper presents empirical evidence by applying a WEF nexus-based composite index to African countries. The WEF Nexus Index^23^ is an innovative policy and decision support tool which can serve as an entry point to inform approaches to addressing water-, energy- and food-related challenges in Africa (and other countries or regions where equitable access to resources is an aspirational goal).

### An African context and the WEF nexus

Africa is undergoing rapid change. Urbanisation, population growth, climate change, and economic shifts are taking place, with infrastructural development being a primary focus of governments, regional development communities, and continental bodies^24^. The prediction is that Africa’s population of 1.1 billion citizens will double by 2050, with more than 80% of that increase occurring in cities and their associated slums^25^. At a continental level, the African Union’s (AU) *Agenda 2063* strategic framework describes the continent’s socio-economic transformation vision over the next fifty years (with 2013 as the base year). *Agenda 2063* envisions, amongst other targets, a “continent with free movement of people, goods, capital, and services and infrastructure connections”^26^.

Water, energy, and food are at the core of Africa’s development agenda. Specifically, *Agenda 2063* envisions, amongst other targets, improved water, energy, and food securities as a prerequisite to unlocking economic development on the continent^27^. This is outlined further in several strategic documents such as the African Water Vision 2025, the *Comprehensive Africa Agriculture Development Programme* (CAADP)^28^ and the *African Bioenergy Framework and Policy*^29^. Together, they outline the AU’s plans for attaining water, energy, and food security. Taking their lead from the AU, regional blocs and member states within Africa have aligned their programs with the *Agenda 2063* strategic documents. Further, they have highlighted opportunities for promoting integrated resource management, planning, and governance. Within the Southern African Development Community (SADC), the WEF nexus has evolved as a means for measuring regional priorities, namely (i) simultaneous WEF securities, (ii) job and wealth creation, (iii) strengthening regional integration, and (iv) sustainable natural resource management^30–33^. The *Revised Regional Indicative Strategic Development Plan 2015–2020* (RISDP), SADC’s blueprint for regional integration, provides an enabling environment for implementing the WEF nexus approach at a regional level. Through a process led by the Global Water Partnership in Southern Africa, sectoral and multi-sectoral frameworks in the form of policies, strategies, plans, programs, and institutional arrangements were developed together with a *SADC Regional WEF Nexus Framework*^19^. The SADC’s focus, which the SADC Council of Ministers has formally endorsed, has allowed for an outcomes-based use of the WEF nexus as a tool for transboundary management of resources, policy formulation, and sustainable development. The WEF nexus has evolved into a people-centric, outcome-based approach. This resonates with the *SADC WEF Nexus Action Plan*, which forms part of the *Regional Strategic Action Plan IV* (RSAP IV)^34^.

While SADC countries are actively committed to the WEF nexus and the SDGs, making development decisions requires an understanding of synergies and trade-offs (which a nexus approach provides), an ability to adapt to a changing climate and economic situations, and subsequent monitoring and reporting. Despite the progress made to date, few initiatives have converted this opportunity into an actionable plan that can inform policymakers. Referring to southern Africa, Schreiner and Baleta^35^, argued that the nexus approach had become part of the current development discourse, observing that there are clear opportunities for sharing resources internationally for the shared benefit of the region. Bullock and Hülsmann^36^ concluded that any future hydro-power development in the zone must be ‘nexus-oriented’. However, Mabhaudhi et al.^24^, explain that there has been a gap between water and energy sector planning regarding policy alignment and technical implementation, which hinders progress towards the SDGs. Water, energy, and food, for example, are often isolated in planning, management, and monitoring.

It has been argued that in the post-colonial age, Africa is following a neoliberal development trajectory, which risks exacerbating the inequalities of the colonial era where access to, and availability of, resources is focused on a select few^37^, thus rendering development unsustainable. While this criticism must be considered, it is the intent and priority of governments on the continent to redress the inequalities of the past. This includes a just transition that yields equitable access to vital resources, including guaranteed freshwater, affordable clean energy, and sufficient affordable, healthy food^38–40^. In several countries, this is expressed as a development goal and human right. For example, South Africa developed a just transition policy (framework, investment plan, partnership) for guiding transitions such as climate action and decarbonisation with minimum social and economic impacts on the livelihoods of the involved vulnerable people and planet^41^. Redressing the history of colonial development in these countries, where the provision of resources was focused on a minority, highlights the imperative of equitable access to water, energy, and food. This is prominent in the SDGs (e.g., 6.1, 6.2, 9.1). Africa is rich in resources, both human and natural. The challenge is to harness these in a sustainable and just way, while addressing the increasing variability of resources as a result of a changing climate, a growing population, land degradation, and urbanisation at a rate that poses a challenge to the state agencies tasked with providing these services.

Sustainable management of resources in Africa requires the generation of new knowledge relevant to Africa, which considers explicitly both the importance of access to resources and the mechanisms enhancing their supply. A nexus approach provides a lens for examining the interaction of these resources with society and each other.

**Figure 1:**
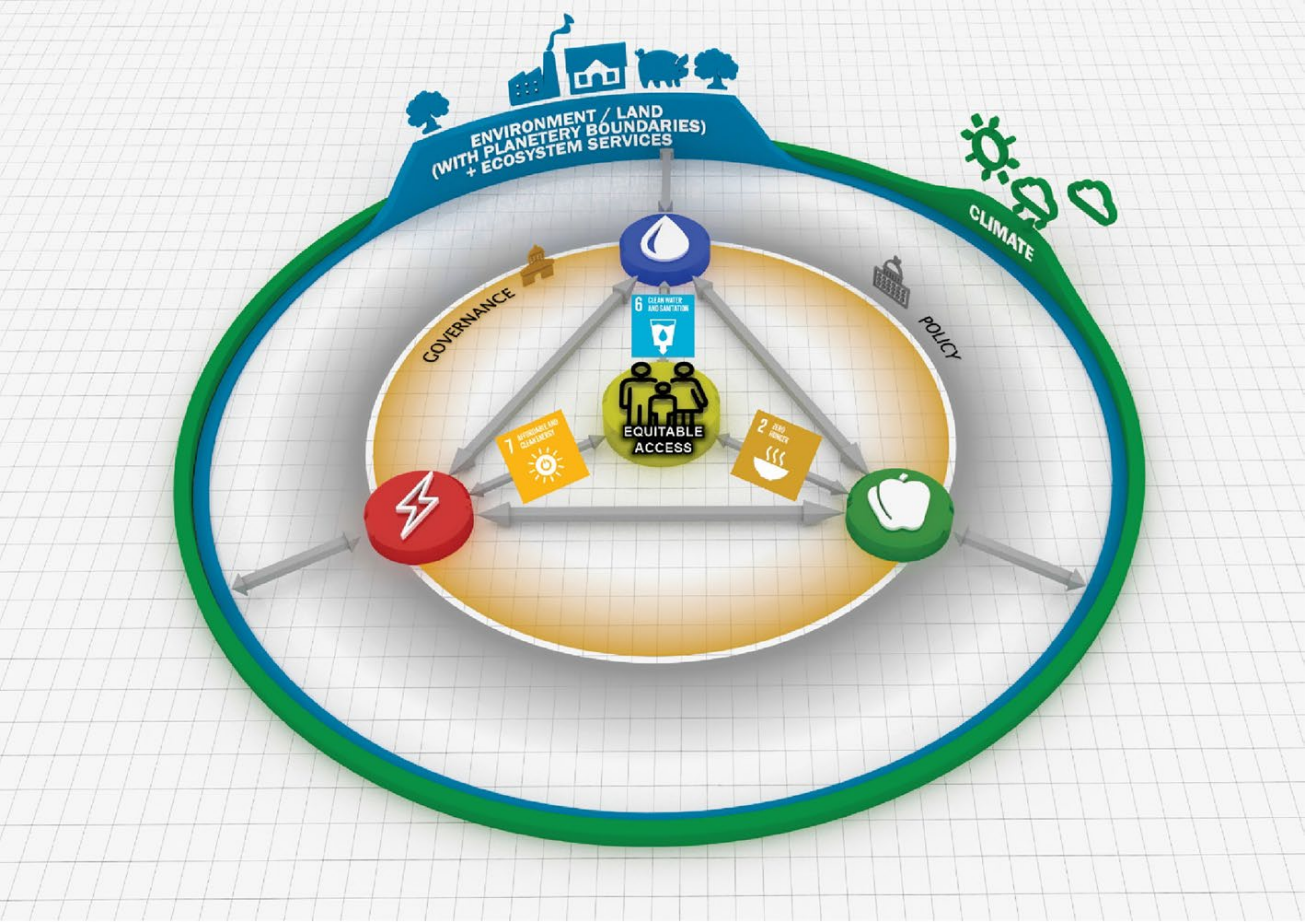
The anthropocentric WEF nexus framework^20^.

Figure 1 presents an anthropocentric WEF nexus framework, which places equity and humanity at its centre, through which the varying aspects within this system are represented^42^. This framework contrasts with many WEF frameworks that emphasise interactions between resources sectors, but do not accentuate the role of society as both a manipulator and beneficiary of the system. This framework is especially applicable to developing regions/countries due to its emphasis on SDGs 2, 6, and 7. This conceptual framework aims to direct the development of tools to address Africa’s policies that promote equitable access to resources, sustainable development, and the safeguarding of the environment and environmental rights.

A lack of access to food could be due to poverty and not necessarily the unavailability of food, hence the importance of other SDGs, such as SDG 8 (amongst others). This implies that any model, innovation, or indicator utilising the WEF nexus framework must incorporate stakeholder engagement, job creation, economics, and/or investor sentiment in the analysis^42^. Therefore, the need for complementary qualitative and quantitative studies is essential^21^.

Humanity, with the accompanying drivers of change that are a focus in the Global South—namely urbanisation, equity, and population growth—are at the centre of the proposed anthropocentric WEF Nexus approach. Simpson^42^ noted that the connections between each of the resource sectors and the centre of the framework is not restricted to the supply of water, energy, and food. Equitable access, denoted by SDGs 2, 6, and 7, is the second portion of the link between the respective resources and humanity. The interdependencies between the three primary components are represented by the direct links between water availability, energy generation, and food production. Water, energy, and food are ultimately, sourced from the environment. The climate influences the natural realm. It is, in turn, influenced by how these resources are obtained or sourced. This supply can be either non-renewable or renewable. Regarding food, it can be produced domestically or imported. All echelons of the system are influenced by policies and governance, which are subject to humanity’s preferences and decision making. People, therefore, drive the global supply chain from the core of this framework, while exerting a significant influence throughout. If we are to obtain all our demands from Earth in the long term, then we must, in turn, govern wisely and develop applicable, cohesive policies^42^. Resource demand management (SDG 15), sustainable supply (SDG 8.4/12.2/12.5), the reduction of greenhouse gases and climate resilience (SDG 13.1), and food waste management are also imperative and indirectly linked to WEF nexus approaches.

The application of WEF nexus planning on the African continent and its regions and nations has received limited attention from research and this can be partly to blame for the slow uptake of the approach^30,43,44^. Building on a previous study by Simpson et al.^23^ which developed the WEF Nexus Index for 181 nations and applied it to analyse SADC countries, this study has sought to assess the opportunities and constraints for integrated resource planning and security of African nations using the WEF Nexus Index and the Human Development Index (HDI) and to recommend potential interventions for improving resource security and sustainability on the continent.

## Methodology

The WEF nexus tool developed by Simpson et al.^23^ was applied to generate country level assessments for all African countries. Furthermore, linkages were sought between the WEF Nexus Index and the HDI to assess the correlation between these two indicators as they assess development from two different perspectives.

### Africa

The African continent is home to approximately 1.3 billion people^45–47^. Africa’s population is relatively young with 60% of the continent’s population under the age of 25^48^, while 70% of sub-Saharan Africa is under the age of 30^49^. Africa faces multiple challenges which undermine universal access to water, energy, food and related resources and these include a cycle of poverty, a rapidly growing population (2.4% per year), rapid urbanisation, inequality, and declining land and agricultural productive capacity due to high levels of land, forest, and soil degradation^50^. It is estimated that in 2021, 490 million people (34%) in Africa lived in extreme poverty, i.e., under the international poverty line of 1.90 PPP$/day^51^. Africa’s rate of urbanisation is the fastest in the world and the urban share of the continent’s population has doubled from 19% to 39% over the last 50 years^52^. Africa is home to the fifteen fastest-growing cities in the world and it will experience a sharp increase in its urban population in the future^53^. Society in Africa is highly unequal with a regional Gini coefficient of around 40%, ranging from 27.6% (Algeria) to 63.3% (South Africa)^51^.

Despite the continent’s vast, but unequally distributed, endowments in water, energy and food resources, Africa’s population, especially those in rural areas have limited access to clean and safe water, affordable and clean energy, and balanced and nutritious diets^30^. Africa is the world’s second-driest continent after Australia^54^. Almost half of the continent’s population lack access to electricity and more than half rely on polluting traditional energy sources such as hard biomass which constitutes 45% of total primary energy demand^55^. The average annual per capita electricity consumption in Africa has remained low and almost constant during the last decade, for example at around 665 kWh per capita in sub-Saharan Africa compared to the global average of approximately 3000 kWh in 2021^55,56^. Smallholder farmers in the dry regions of Africa depend on rain-fed agriculture for food production, income generation, and livelihoods and are vulnerable to climate variability and frequent natural disasters^57^. The security of water, energy, and food resources in Africa is exacerbated by pandemics (e.g., COVID-19), adverse effects of climate change (e.g., floods and droughts), and geopolitical conflicts (e.g., the Russian-Ukrainian war) ^45,46,58^.

### The WEF Nexus Index

A composite index is formed when distinct indicators are combined into a specific index on the basis of a systemic, underlying context ^59,60^. Nardo et al.^61^ presented selected benefits and disadvantages associated with composite indicators (Table 1). Although Saltelli^62^ argued that “the use of composite indicators is very much the subject of controversy, pitting aggregators against non-aggregators”, a benefit of an index is that a single number can represent a complex, integrated system in a consolidated way. The index for a basin, country, or region can be compared to an aspirational value or compared with other countries or regions, or to values for the same nation at different time scales (either historical or future) to determine progress and/or trends. This serves as a knowledge-generating and decision-support tool, which can be used to inform policy. An index, therefore, serves as an entry point into the analysis, rather than an end in itself and can be a valuable tool for assisting in the resolution of complex development challenges.

**Table 1:** Pros and cons of composite indicators after^61^.

Pros of composite indicators	Cons of composite indicators
Summarise complex or multi-dimensional issues, in view of supporting decision-makers	May send misleading policy messages if they are poorly constructed or misinterpreted
Are easier to interpret than trying to find a trend in many separate indicators	May invite the drawing of simplistic policy conclusions if not used in combination with the indicators
Facilitate the task of ranking countries on complex issues in a benchmarking exercise	May lend themselves to instrumental use (e.g., be built to support the desired policy) if the various stages (e.g., selection of indicators, choice of model, weights) are not transparent and based on sound statistical or conceptual principles
Assess the progress of countries over time on complex issues	The selection of indicators and weights could be the target of political challenges
Reduce the size of a set of indicators or include more information within the existing size limit	May disguise serious failings in some dimensions of the phenomenon, thus increasing the difficulty of identifying the proper remedial action
Place issues of countries’ performance and progress at the centre of the policy arena	May lead to wrong policies if dimensions of performance that are difficult to measure are ignored
Facilitate communication with ordinary citizens and promote accountability	

**Figure 2:**
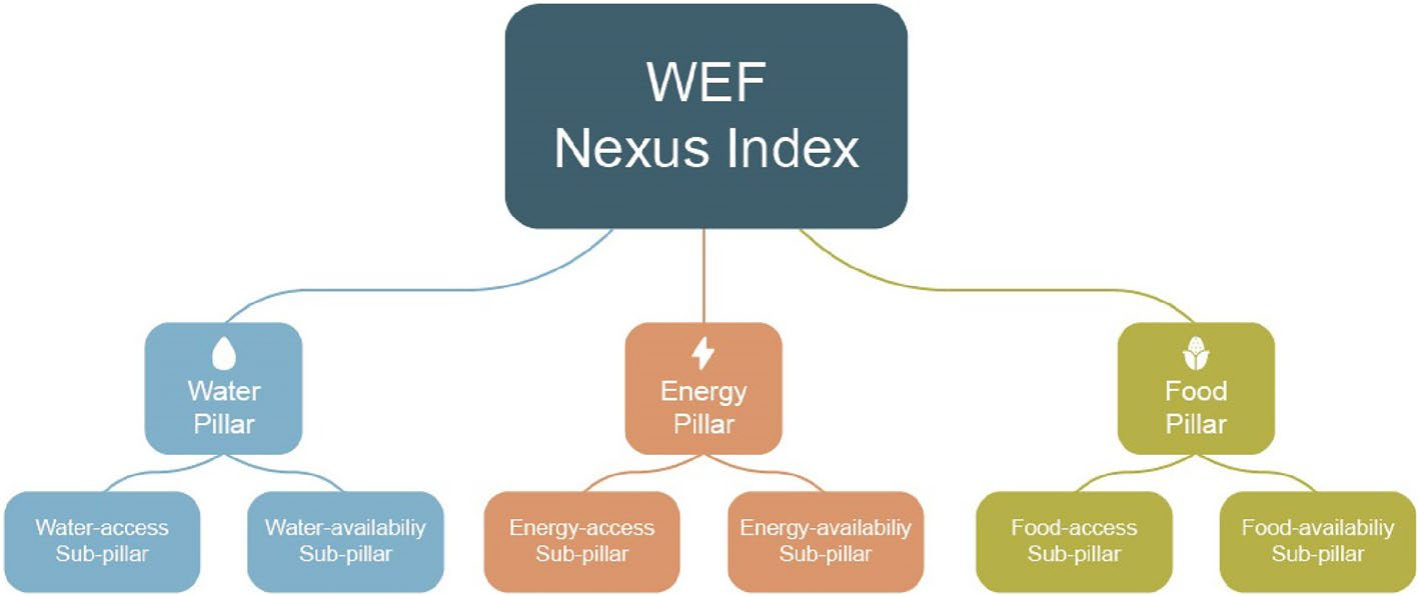
Pillars and sub-pillars associated with the WEF Nexus Index^23^.

Simpson et al.^23^ undertook an assessment of 87 globally applicable (and available) water-, energy-and food-related indicators and subsequently selected 21, which were used to develop the WEF Nexus Index. The selection criteria included relevance, added value, data availability, and reliability, together with a correlation analysis to identify possible aggregation issues or double-counting^23^. Correlation analysis was necessary for dealing with redundancy in case(s) of multicollinearity. Missing data were imputed where appropriate or necessary in accordance with the *Joint Research Centre’s Competence Centre on Composite Indicators and Scoreboards* (JRC:COIN) guidelines as outlined in Simpson et al.^23^. The indicators were selected, normalised by the min–max method, transformed into a uniform scale of 0 – 100, gap-filled by shallow imputation, and treated for outliers, weighted equally and aggregated as an arithmetic mean as fully described in Simpson et al.^23^. To ensure comparability, only globally available indicators were utilised in the construction of this index. The framework presented in Fig. 1 serves as the foundation for the index. It explicitly considers both access to and availability of resources (Fig. 2).

The access sub-pillars are specifically intended to address the equity component of the aspirational SDGs. Simpson et al.^23^ explain that the aim of developing the WEF Nexus Index is to create a country-level, quantitative, integrated measurement of resource securities related to access to and availability of water, energy, and food. It provides a measure of the degree of achievement of SDGs 2, 6, 7, and 13. It is a tool, lens, and means for initiating integrated resource management, not an end in itself, and is supported by a strong emphasis on data visualisation and representation. In this regard, the visualisation website presented with the WEF Nexus Index (https://www.wefnexusindex.org/) can be utilised to catalyse or complement WEF Nexus assessments. A high index value indicates that a nation has achieved a relatively good, integrated resource management and security related to access to and availability of water, energy, and food. The index can also be utilised for determining national progress regarding integrated resource management. The index has been calculated globally for individual countries—the results for Africa are the focus of this paper, and readers are referred to Simpson et al.^20^ and Simpson et al.^23^ for detailed information on how the WEF Nexus was developed.

### The Human Development Index

The HDI is an established composite summary index that measures key dimensions of human development, deviating from the prior sole focus on economic growth—it emphasises dimensions of people and their capabilities related to education, health, and income per capita^63,64^. The three key dimensions (dimension indices) of human development include a long and healthy life (indicator is life expectancy at birth), access to education (indicators are expected years of schooling of children at school-entry age and mean years of schooling of the adult population), and a decent standard of living (indicator is Gross National Income per capita adjusted for the price level of the country)^63,64^. The HDI seeks to shift the focus from the usual economic statistics to human outcomes and the higher a country's human development, the higher its HDI value^65,66^. On the other hand, the WEF Nexus Index provides an alternative lens on development, i.e., one that is centred on integrated resource management. Thus, this study also sought to ascertain the degree of correlation and the reason for any outliers between these two indices (WEF Nexus Index and HDI) for informing their complementarity and to assess the unique contribution the WEF Nexus Index could make to the sustainable development discourse.

### Data sources

The WEF Nexus Index was initially developed using national level data for 2019 from open databases such as those by provided national statistical offices, government departments, non-governmental organizations and international organizations such as the World Bank, International Energy Agency (IEA), and Food and Agriculture Organisation of the United Nations (FAO), and World Health Organization (WHO)^23^. The inclusion–exclusion criteria for such data and indicators included availability of valid data in at least 65% of the (i) countries, and (ii) indicators. The HDI data was obtained from the Human Development Reports by United Nations Development Programme (UNDP).

## Results and discussion

**Figure 3:**
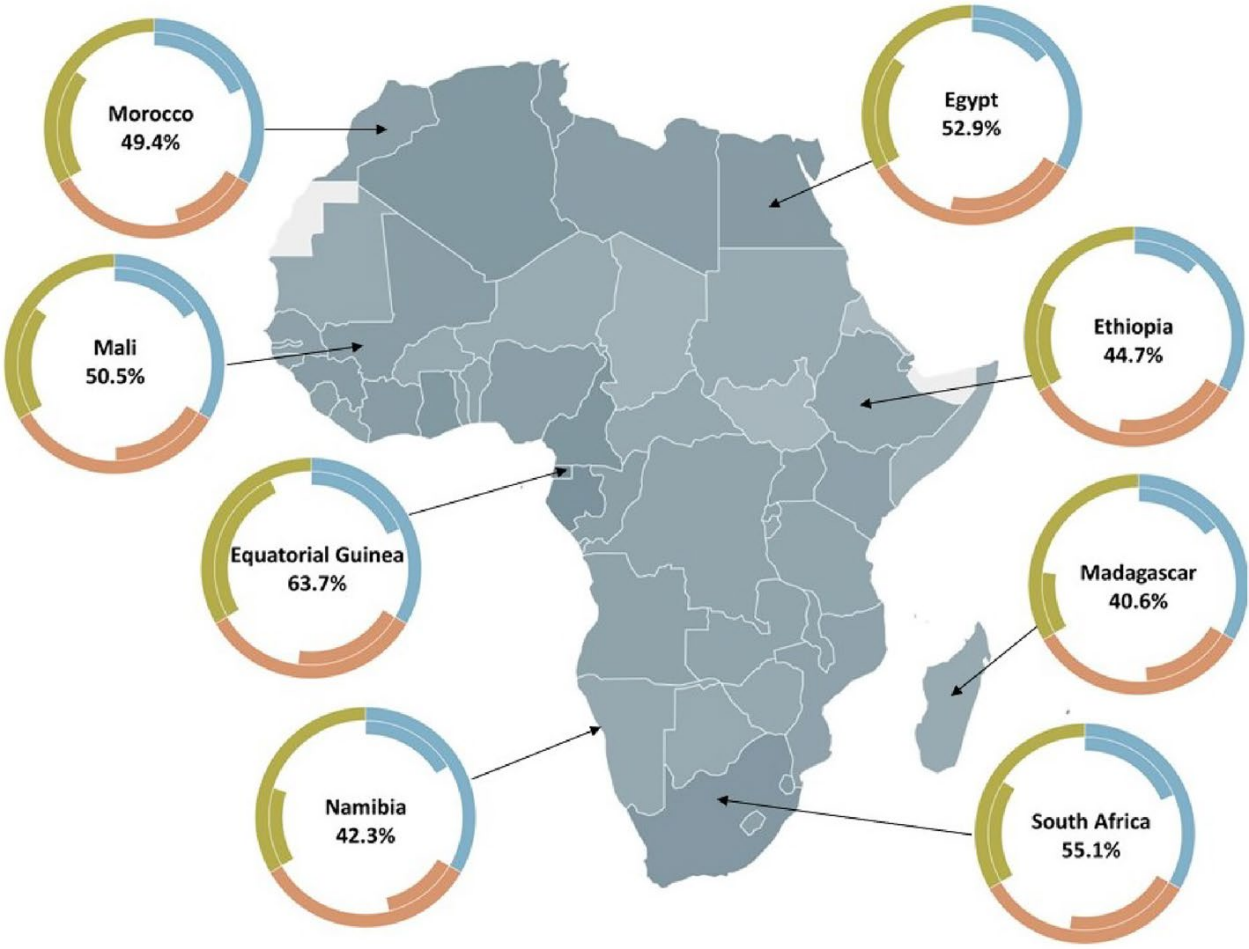
Map of Africa indicating the WEF Nexus Index per nation, with specific countries featured in doughnut plots. The outer bounds of the doughnut plots indicate the maximum possible score (100%) for the water (blue), energy (orange), and food (green) pillars associated with the WEF Nexus Index. The inner bounds indicate the proportion of that score achieved by the country associated with that doughnut plot.

Sufficient data (2019) were available to calculate the WEF Nexus Index for 54 African nations. The index values for selected countries evaluated are presented in a map of the continent (Fig. 3). Annexure 1 presents the WEF Nexus Index, pillar, sub-pillar values, and ranks for each African country. The five highest-ranking African countries in terms of the WEF Nexus Index are Equatorial Guinea, Seychelles, Gabon, Cabo Verde, and São Tomé and Principe, with global ranks of 44th, 64th, 79th, 83rd, and 85th, respectively, out of the 181 nations assessed. The assessment of Africa’s relatively low scores in terms of the WEF Nexus Index does not represent a negative narrative. Instead, it presents an opportunity to undertake a ‘differential diagnosis,’ as Sachs^67^ proposed, to understand the underlying challenges and possible remedies for the achievement of long-term aspirational development goals.

### WEF nexus scores for Africa

The indicator values associated with the calculation of the WEF Nexus Index are presented in Annexure 2. The following section provides a high-level differential diagnosis to illustrate how the index can be used to provide insight into development opportunities for the continent. The 21 indicator metrics used in the determination of the WEF Nexus Index include, inter alia, annual freshwater withdrawal, average precipitation in depth, average value of food production, cereal yield, average dietary energy supply adequacy, average protein supply, access to electricity and per capita electric power consumption^23^. In terms of *annual freshwater withdrawal,* Egypt, Libya, Sudan, and Mauritania withdraw 4100%, 823%, 673%, and 337% of their annual internal freshwater resources, respectively. Egypt has one of the highest freshwater withdrawal ratios of the 54 nations assessed in Africa. This indicates that either water is generated outside of the country’s borders and/or imported. These withdrawal rates and high water dependencies represent a significant risk to their long-term development and are a potential source of conflict. In contrast, many other African countries withdraw less than 10% of their internal freshwater resources, as shown by the low values for that indicator, especially in Central Africa^47^. This disparity in water availability across Africa is supported by the indicator, *average precipitation in depth*, where values vary between 51 mm per year (Egypt) and 3200 mm per year (São Tomé and Principe), with the median value for African nations being 955 mm per year. Where countries have a high degree of freshwater availability, but low access levels due to economic water scarcity, this provides clear guidance for policymakers, i.e., the water is there, and policy and implmentation should focus on mechanisms work towards getting it to the people, for example in the Central African countries. Several African nations generate hydro-power which is exported to neighbouring countries, i.e., water is used for energy, which is sold for economic development. Where the opposite is true, i.e., high levels of access in areas where water is a scarce commodity, the policymakers must focus on leak detection, correct allocations, and water demand management to a greater degree, for instance in the North African region.

The *average value of food production* is measured in I$ (An international dollar (I$) could purchase, in a specific nation, a comparable number of goods and services that a US$ would obtain in the United States. This term is often used together with Purchasing Power Parity data ^68^.) per capita per annum. Only six African nations have average food production values that exceed the global median value, namely, Tunisia, Ghana, Cote d’Ivoire, Morocco, Mali, and Cameroon. The remaining three WEF Nexus Index “food-accessibility” indicators, i.e. *average protein supply*, *cereal yield*, and *average dietary energy supply adequacy* are crucial targets for policy and development interventions in Africa. Vitousek et al.^69^ noted that in the twentieth century, “Unlike most regions of the world, crop yields have not increased substantially in sub-Saharan Africa.”

Overall, African countries generally have low crop yields relative to their potential (the median cereal yield for the African countries assessed is just over one-third of the median for the 181 nations). They also receive relatively little income for the crops they produce. This represents a high yield gap, with the dominance of a few staple crops. This scenario highlights challenges that many African countries face in seeking to achieve their full agricultural potential, a problem that is systematically being addressed through sound implementation of initiatives such as the CAADP.

A substantial challenge for many African countries is how to beneficially utilise the available freshwater resources to sustainably enhance the cultivation and production of food and other agricultural products. The WEF Nexus Index and its constituent indicators suggest an opportunity for improving water supply and developing agricultural potential. However, a more detailed analysis is needed to understand the context in which this could occur. The question of how to positively utilise the water resources in many African nations to enhance food production is especially pertinent since 95% of sub-Saharan Africa’s food production is rainfed, i.e. by green water^70–72^. Thus, there are opportunities in terms of drought-proofing rainfed farming^73^ and for a growth in irrigation development^74^. These two pathways are key pillars in the African Union’s *Framework for Irrigation Development and Agricultural Water Management* (AU-IDAWM) which is yet to be operationalised at sub-continental scales^75^.

Furthermore, Mueller et al.^76^ explain that global yield variability is greatly controlled by fertiliser use, irrigation, and climate while simultaneously emphasising the importance of soil characteristics and agricultural management practices. Consequently, any policy decision or action to improve water supply for agriculture must occur in tandem with efforts to enhance nutrient balances (through appropriate fertiliser addition), access to markets, and agricultural training and research. Lu and Tian^77^ note that “Africa is still characterised by low nutrient input along with expanding cropland areas.” Therefore, more affordable access to fertilisers is crucial to improve crop yields and productivity on this continent. Van der Zaag^74^ calls for “location-specific interventions that are aimed at enhancing farmers’ capacity to buffer water variations and address nutrient deficits.” The former of these can potentially be achieved by developing the third of the WEF nexus sectors, i.e., energy. Access to affordable energy will facilitate “finding ways to enhance farmers’ control over water” ^74,78^ for agricultural production through the increased use of both irrigation and rainwater (e.g., through rainwater harvesting, improved soil water management, and access to natural storage systems).

Beyond the water link, analysing energy patterns from the WEF Nexus Index, highlights that approximately 31% of African nations lie below the global 10th percentile value for *access to electricity*. Further, only one country in Africa has a per capita *electric power consumption* that exceeds the median value obtained in the global assessment, namely, South Africa. Energy is a vital enabler of economic activity, including the agricultural sector. The agricultural sector utilises 71% of blue water globally^79^, over 85% in Africa^80^, and between 70 and 80% in southern Africa^81^. Effective utilisation of blue water pumping and distribution using energy should accompany any agricultural expansion. For example, agricultural post-harvest processing is a major limitation in many African agricultural systems and requires a steady supply of energy. This is the case for both large scale and smallholder farmers. Indeed, many current studies highlight the positive benfits of enhanced energy supply to smallholder farmers^27,82–86^. However, there is a significant scope to develop the energy sector in Africa. The four primary sources of electricity in Africa are natural gas, coal, hydro-power, and oil^87^.

This WEF Nexus Index assessment suggests that national policy and development for providing clean and affordable energy to people, particularly in the agricultural sector, will be an enabler of economic growth in many African countries. Examples of such projects are the mega-solar projects that are planned in Botswana and Namibia that could add up to 5000 MW of new solar power over the next two decades^88^—well as and implementation of the Grand Inga Dam Hydropower Project. Having highlighted this gap through the WEF Nexus Index, a more detailed analysis shows that there are many opportunities for smaller distributed systems to supply and enable access to energy in Africa. These include solar pumps for smallholder farmers^89–91^ and off-grid, solar, domestic electricity systems^92,93^.

While many African countries have a high level of renewable electricity output as a proportion of their total electricity output (e.g., the Central African Republic, the Democratic Republic of Congo, Ethiopia, Lesotho, Namibia, and Zambia), these are primarily hydro-electric schemes, and the actual quantum of electricity generated is generally low, sometimes below the installed capacity, for example in Zimbabwe. In contrast, other nations (e.g., Algeria, Botswana, Niger, Seychelles, South Africa, South Sudan, and Tunisia) have a level of renewable electricity output that is less than five percent of the total electricity output.

Although South Africa and Libya are notable exceptions, most African nations do not contribute significantly to anthropogenic climate change. However, the development of renewable energy and agriculture will require related enabling policies, which facilitate the development of land, extension services, markets, supply chains, and international trade. Further, it will be necessary to recognise that traditional development approaches associated with mega-projects, e.g., large scale irrigation developments, are not always applicable (successful) or appropriate when compared to small-scale projects such as farmer-led irrigation^94–97^.

### Human development and the WEF Nexus Index

**Figure 4:**
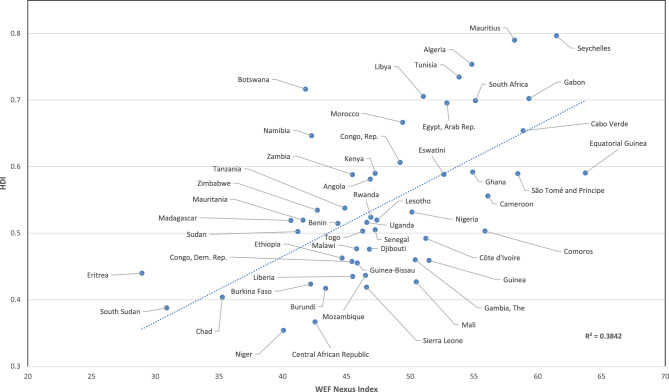
Plot of the Human Development Index versus the WEF Nexus Index for 54 African countries.

Figure 4 presents a plot of the HDI against the WEF Nexus Index for the 54 African nations under assessment. The trendline of the data, which passes between Chad and Gabon, provides an indication of which countries have higher (above the trendline) or lower (below the trendline) human development relative to their water-, energy-, and food-resource base and service delivery. Nations such as Mauritius, Botswana, and Namibia have relatively high levels of human development relative to the WEF Nexus Index. Policy lessons from nations that plot above the trendline, particularly the most extreme outliers, could be applied to other nations that currently lie below the trendline. What follows is an assessment of selected countries based on four clusters of the WEF Nexus Index versus HDI configurations, i.e., high-high, low-low, low–high, and medium–low.

### Gabon (relatively high WEF Nexus Index, relatively high HDI)

Gabon has the third-highest WEF Nexus Index of the 54 African countries assessed and has a relatively high HDI (0.7). In terms of water, the 2019 values indicate that 87.5% of the Gabonese population has access to at least basic drinking water standards. The annual freshwater withdrawals in this nation constitute 0.085% of the internal resources, indicating that the country has significant water resources that are not currently exploited. The renewable internal freshwater resources are 87,433 cubic metres per capita, which is the highest volume for any of the African nations. This value exceeds the 90th percentile for the 181 countries assessed in the global WEF Nexus Index determination (36,886 cubic metres per capita). Thus, it is suggested that Gabon seeking to effectively utilise its available water resources to achieve universal access to basic drinking water standards with its available resources is feasible.

A total of 91.4% of Gabon’s citizens have access to electricity and 82% of the nation’s final energy consumption is from renewable sources. However, carbon dioxide (CO_2_) emissions in Gabon amount to 2.8 metric tons per capita, which exceeds the median value (2.4 metric tons per capita) for the global WEF Nexus Index assessment. This level of CO_2_ emissions is amongst the highest for African nations.

The *prevalence of undernourishment* and *children under five years of age affected by wasting* in Gabon are 9.4% and 3.4%, respectively. The level of undernourishment is equal to the 60th percentile value for the global assessment. The *average protein supply* within the Gabonese population is 58 g/capita/day, which is less than the 30th percentile for the 181 nations. The *average value of food production* in this nation is I$ 136 per capita, which is also less than the 30th percentile value for the global assessment.

### Chad, South Sudan, and Eritrea (low WEF Nexus Index, low HDI)

In contrast, Chad, South Sudan, and Eritrea have WEF Nexus Index values of 35.3, 30.9, and 29, respectively. These three African countries rank as the three lowest nations in terms of the global HDI assessment. The populations in these countries have low levels of access to basic drinking water services, safely managed sanitation services, and electricity. In Chad, the indicators *prevalence of undernourishment* and the *percentage of stunted children under five years of age* are 39.7% and 39.9%, respectively. In these countries, the WEF Nexus Index and HDI are aligned, but the WEF Nexus Index provides more insight as to the effect that poor access and availability to these critical livelihood resources of water, energy and food has on the populations.

### Botswana and Namibia (low WEF Nexus Index, relatively high HDI)

Botswana and Namibia are examples of two nations that have relatively high HDI values when compared to their WEF Nexus Index values, i.e., they are the two countries that plot furthest above the regression line in Fig. 4. Botswana and Namibia rank 171st and 169th in the global WEF Nexus Index determination, respectively. Both nations have low annual freshwater withdrawals, which is probably due to the highly seasonal and localised nature of their water resources. However, their relatively high education and literacy values raise the HDI. Policies in this regard could be instructive to other African nations.

### Niger (medium WEF Nexus Index, low HDI)

Niger is a nation that lies well below the regression line in Fig. 4 and ranks 176th out of 181 nations in terms of the WEF Nexus Index, with a value of 40.1. This implies that Niger could potentially enhance its use of natural resources for the benefit of its population’s living standards raising both HDI and WEF Nexus Index scores. However, a key challenge is that this landlocked nation has an average precipitation of only 151 mm per annum. This can be contrasted with other countries such as the Democratic Republic of Congo, Congo, Gabon, Guinea, Guinea-Bissau, and Sierra Leone, which all receive over 1500 mm per year, on average. Notwithstanding this low rainfall in Niger, the country attains a relatively high average value of food production compared to other African countries. Both *electric power consumption* and *access to electricity* are, however, low, at 51 kWh per capita and 16.2%, respectively.

Based on this assessment, it is evident that the correlation between the HDI and the WEF Nexus Index is moderate at 0.38. Interestingly, the correlation between the HDI and the various sub-pillars (water access and availability, energy access and availability, and food access and availability) indicate that there is a medium to strong correlation between the HDI and both the water-access and energy-access sub-pillars. These correlations support the argument that access to water and energy greatly enhance human development.

### Summary

A primary goal of the WEF nexus community is to support development that is equitable, just, and sustainable. To do so, it must identify policy proposals and innovative development projects that meet these criteria. These should be informed by both qualitative and quantitative assessments and include representative stakeholder engagement. WEF nexus-based studies, policies, and projects must be focussed on the direct and indirect achievement of SDGs 1, 2, 6, 7, and 13, both in terms of access and availability, to ensure distributional justice, especially in the African context. Such actions, combined with broad public participation, can have a ripple effect on other SDGs such as SDGs 5, 10, and 17, thereby reducing inequalities and building partnerships to attain these aspirational goals.

The WEF Nexus Index can serve as an entry point and catalyst into a developmental ‘differential diagnosis,’ as has been demonstrated for Africa in this paper. The constituent values of the water, energy, and food pillars that make up this multi-centric index provide a second level of assessment for a WEF nexus study. An analysis of the energy sub-pillars for the African nations in this study has indicated that a limitation to enhancing agriculture in most of these countries is the lack of access to energy. This is supported by the moderate correlation between the HDI and the energy-access sub-pillar. This is important as it contrasts with widely held perceptions that water is the principal limiting factor in enhancing agricultural production. Policies that balance the drought-proofing of rainfed agriculture and irrigation development are needed. This conclusion should inform the CAADP goals regarding the expansion of areas under irrigation in many African nations, as well as the strengthening of the supply chains associated with the entire agricultural sector. This assessment must be balanced with other policy decisions, such as facilitating more affordable and easily accessible agro-inputs for use on this continent. Access to markets and research that are linked to extension work (i.e., farmer training) are also essential.

Increased access to energy can also enhance improved domestic water and sanitation services. This analysis provides an additional policy thrust for the continent’s energy plan (which has tended to link energy to the industrialisation agenda) and highlights the opportunity for policies that focus on the development of energy from renewable sources. The need to develop clean, affordable, renewable energy as an *enabler* for agricultural development should also be incorporated into regional plans such as SADC’s RISDP, RSAP IV, and the Regional WEF Nexus Framework.

This analysis of the WEF nexus in Africa illustrates both strengths and weaknesses of the WEF Nexus Index. The composite indicator highlights where the need for development to support human well-being lies. However, it also illustrates that care must be taken in the interpretation of the index values. At a macro level, it could be concluded that African countries should invest heavily in large-scale water storage and energy supply infrastructure. However, detailed analysis provides a more nuanced message, which indicates that smallholder agriculture systems, which dominate sub-Saharan African food production, may require more balanced solutions, such as local storage and small-scale energy production. The identification of adjacent realities, which can be missed when only global indicators are considered, is required. Examples in this regard are decentralised energy, soil fertility, the use of high-yielding crop varieties, and applying the best practices in agronomic management.

The assessment of Africa’s relatively low scores in terms of the WEF Nexus Index does not represent a negative narrative. Instead, it provides provides high-level insights and an entry point to understanding the underlying challenges, through which more detailed analyses can lead to identified solutions and policies. Many African countries are trapped in an environment that could be termed a ‘poverty-unemployment-inequality nexus’ (due to the interlinkages that exist between these ‘wicked’ problems)^1,2,98–101^. Africa’s abundant resources, such as minerals, metals, land, water resources, relatively warm temperatures, high solar radiation, and resilient people provide opportunities to address this nexus and achieve an equitable and ethical benefit, such that we ‘leave no one behind.’

### Limitations

The main limitations of the index-based approach applied in this study were highlighted in Table 1. A major challenge lies in potential generalization of the situation which may mask some underlying dynamics of the WEF system. The quantitative nature of physical indicators in the WEF Nexus Index ignores the political and social aspects of the WEF nexus and we recommend that these are included in further studies or improvements of the tool. However, the WEF Nexus Index is a key entry point in nexus analysis and planning, and we recommend its simultaneous use with the indicators for informing practice and policy.

## Conclusions and recommendations

This study utilised the WEF Nexus Index to analyse the WEF nexus in 54 African nations for whom sufficient data was available in 2019. Generally, African countries are performing sub-optimally in the WEF Nexus Index, appearing from the 44th rank in the global list. These sub-optimal performances are a key entry point for identifying challenges and planning intervention strategies for the continent and its member states. Deeper analysis at subnational levels and scales may (i) reveal spatial variations in dynamics of the WEF nexus at local scales, and (ii) inform potential for improvements in policy and practice within and between countries. At sector and indicator levels in the continent, variations exist in the security of the WEF nexus which necessitates contextualised analysis for countries and their subnational scales. Even at pillar and indicator level, African countries are performing poorly in all three fronts of resource security: water, energy and food.

African countries with high water availability and low water access need to invest in the appropriate water treatment, distribution and supply infrastructure, while those with scarce water resources need to optimise on water use efficiency and productivity. Potential for enhancing energy security lies in the implementation of planned power generation projects, especially renewables across large, medium and small scales. A low 'hanging fruit' for boosting power generation lies in optimising production in existing power plants through repair, maintenance and rehabilitation. Agriculture is the mainstay for sustaining livelihoods and catalysing economic growth in Africa and its development for production of food and fibre is inextricably connected to water and energy security. Recommended actions for enhancing food security in Africa include climate smart agriculture (CSA), agricultural water management, and soil fertility (nutrient) management for improved yields in line with the African Union’s *Framework for Irrigation Development and Agricultural Water Management* (AU-IDAWM). However, in all cases, planning and implementation of interventions seeking security of water, energy and food resources need to be conducted from a nexus perspective to optimise synergies and minimize trade-offs.

The moderate correlation between the HDI and the WEF Nexus Index implies that food and nutrition security and service delivery in terms of access to freshwater, safe sanitation, and electricity greatly catalyse human development.

Further analysis is necessary to assess the scalability of the WEF Nexus Index. For example, what additional local-scale indicators are required to ensure that its interpretation is more context-specific; or which policy outcomes meet the stated primary objective, i.e., supporting human well-being? In so doing, the warnings of a “blue water bias”^102^ and the risks of following an inappropriate normative development agenda^37^ will be considered.

### Supplementary Information

Supplementary Information 1.

Supplementary Information 2.

## Data Availability

The dataset presented in this study can be found in an online repository at: http://dx.doi.org/10.17632/2krwdc8n8d.1 and in the Supplementary Material.
