# Supplementary material for: An African perspective on the Water-Energy-Food nexus
Source: Sci Rep. 2023 Oct 6;13:16842. doi: 10.1038/s41598-023-43606-9 (PMC10558539; doi:10.1038/s41598-023-43606-9)
Supplement: Supplementary file 1 — Supplementary Information 1. [file 41598_2023_43606_MOESM1_ESM.pdf]

Annexure 1: Sub-pillar, pillar, WEF Nexus Index values and ranks per African nation

Country	Country code	Water-Access Sub-pillar	Water-Availability Sub-Pillar	Energy-Access Sub-Pillar	Energy-Availability Sub-Pillar	Food-Access Sub-Pillar	Food-Availability Sub-Pillar	Water Pillar	Energy Pillar	Food Pillar	WEF Nexus Index	Rank
Equatorial Guinea	GNQ	41.8	68.4	56.8	-	79.4	-	55.1	56.8	79.4	63.7	44
Seychelles	SYC	78.0	71.5	63.2	-	78.1	14.8	74.7	63.2	46.4	61.5	64
Gabon	GAB	41.8	74.4	81.5	52.9	79.6	25.7	58.1	67.2	52.6	59.3	79
Cabo Verde	CPV	68.7	43.4	70.0	-	81.0	20.1	56.0	70.0	50.6	58.9	82
São Tomé and Principe	STP	41.4	88.9	56.4	-	80.9	26.5	65.2	56.4	53.7	58.4	85
Mauritius	MUS	84.2	60.9	69.3	12.6	85.4	36.6	72.6	41.0	61.0	58.2	89
Cameroon	CMR	39.1	69.5	70.9	50.6	78.0	28.4	54.3	60.7	53.2	56.1	98
Comoros	COM	42.1	58.6	74.9	-	68.3	16.4	50.3	74.9	42.4	55.9	99
South Africa	ZAF	71.2	39.6	54.7	60.6	68.6	35.9	55.4	57.7	52.3	55.1	108
Ghana	GHA	41.0	53.2	70.8	50.8	82.9	30.8	47.1	60.8	56.8	54.9	110
Algeria	DZA	73.6	28.7	63.7	53.4	73.3	36.4	51.1	58.5	54.8	54.8	111
Tunisia	TUN	78.2	26.4	67.4	35.3	74.1	41.7	52.3	51.3	57.9	53.8	117
Egypt, Arab Rep.	EGY	74.7	10.1	67.4	54.2	62.5	48.3	42.4	60.8	55.4	52.9	122
Eswatini	SWZ	53.9	38.6	66.7	-	71.9	17.9	46.3	66.7	44.9	52.6	124
Guinea	GIN	30.3	70.2	57.0	-	71.1	23.4	50.3	57.0	47.2	51.5	126
Côte d'Ivoire	CIV	38.5	59.2	61.0	47.0	73.5	28.1	48.9	54.0	50.8	51.2	129
Libya	LBY	78.8	19.6	71.2	54.5	49.8	32.2	49.2	62.9	41.0	51.0	130
Mali	MLI	47.4	47.3	49.5	-	74.8	34.5	47.3	49.5	54.6	50.5	134
Gambia, The	GMB	44.8	45.3	56.6	-	75.0	23.9	45.1	56.6	49.5	50.4	135
Nigeria	NGA	38.2	56.1	62.4	50.3	69.2	24.6	47.2	56.3	46.9	50.1	136
Morocco	MAR	73.6	36.0	69.7	6.2	74.8	35.9	54.8	38.0	55.4	49.4	139
Congo, Rep.	COG	31.1	76.4	62.5	50.4	63.2	11.6	53.8	56.4	37.4	49.2	141
Lesotho	LSO	43.2	46.2	53.5	-	73.3	14.6	44.7	53.5	43.9	47.4	144
Kenya	KEN	40.0	41.5	69.9	41.6	74.1	16.4	40.8	55.8	45.2	47.3	145
Senegal	SEN	53.9	46.4	56.2	23.8	80.4	22.8	50.1	40.0	51.6	47.3	146
Rwanda	RWA	44.3	51.1	53.0	-	66.1	14.0	47.7	53.0	40.1	46.9	147
Angola	AGO	30.5	61.2	51.0	50.7	69.4	18.4	45.9	50.9	43.9	46.9	148
Djibouti	DJI	59.5	42.2	51.2	-	56.5	20.2	50.9	51.2	38.3	46.8	149
Uganda	UGA	30.4	55.4	57.9	-	63.2	14.8	42.9	57.9	39.0	46.6	150
Sierra Leone	SLE	21.8	76.7	47.1	-	64.5	22.2	49.2	47.1	43.4	46.6	151
Mozambique	MOZ	33.9	60.4	54.9	51.1	63.8	14.7	47.2	53.0	39.3	46.5	152
Togo	TGO	28.4	51.5	62.7	40.2	75.1	19.8	39.9	51.4	47.5	46.3	154
Guinea-Bissau	GNB	38.7	60.8	42.6	-	69.4	21.0	49.8	42.6	45.2	45.8	155
Malawi	MWI	44.0	47.1	48.3	-	71.5	15.7	45.6	48.3	43.6	45.8	156
Liberia	LBR	26.2	78.3	45.3	-	61.0	16.9	52.3	45.3	38.9	45.5	158
Zambia	ZMB	39.2	56.9	58.6	47.5	56.5	14.1	48.0	53.1	35.3	45.5	159
Congo, Dem. Rep.	COD	21.6	74.0	55.1	49.2	68.4	4.3	47.8	52.2	36.3	45.4	160
Tanzania	TZA	33.5	52.9	50.8	44.7	68.3	19.0	43.2	47.8	43.7	44.9	162
Ethiopia	ETH	16.0	51.8	68.4	47.1	69.3	15.3	33.9	57.7	42.3	44.7	163
Benin	BEN	41.6	51.1	44.3	26.5	77.1	25.2	46.4	35.4	51.2	44.3	164
Burundi	BDI	38.8	50.6	47.1	-	67.0	9.6	44.7	47.1	38.3	43.4	165
Zimbabwe	ZWE	49.7	39.3	55.7	43.7	57.3	10.7	44.5	49.7	34.0	42.7	167
Central African Republic	CAF	28.5	68.3	50.0	-	46.1	12.2	48.4	50.0	29.2	42.5	168
Namibia	NAM	54.4	42.8	60.3	16.4	65.2	14.4	48.6	38.4	39.8	42.3	169
Burkina Faso	BFA	39.2	41.1	37.3	-	73.7	24.5	40.2	37.3	49.1	42.2	170
Botswana	BWA	55.2	38.5	48.0	32.0	59.9	17.1	46.8	40.0	38.5	41.8	171
Mauritania	MRT	47.3	19.5	42.4	-	68.5	29.5	33.4	42.4	49.0	41.6	172
Sudan	SDN	37.2	24.5	54.7	50.4	59.6	20.8	30.9	52.5	40.2	41.2	173
Madagascar	MDG	23.6	65.3	46.2	-	49.4	13.1	44.5	46.2	31.3	40.6	175
Niger	NER	27.7	32.9	35.1	50.0	70.8	23.8	30.3	42.6	47.3	40.1	176

Somalia	SOM	11.8	29.1	54.2	-	67.1	7.6	20.4	54.2	37.4	37.3	178
Chad	TCD	18.4	44.2	39.4	-	55.5	14.5	31.3	39.4	35.0	35.3	179
South Sudan	SSD	24.4	52.8	24.2	50.0	22.7	11.2	38.6	37.1	17.0	30.9	180
Eritrea	ERI	2.3	29.3	42.1	38.8	58.5	2.7	15.8	40.5	30.6	29.0	181
