# Supplementary material for: An African perspective on the Water-Energy-Food nexus
Source: Sci Rep. 2023 Oct 6;13:16842. doi: 10.1038/s41598-023-43606-9 (PMC10558539; doi:10.1038/s41598-023-43606-9)
Supplement: Supplementary file 2 — Supplementary Information 2. [file 41598_2023_43606_MOESM2_ESM.pdf]

Annexure 2: Indicator data utilised in the calculation of the WEF Nexus Index for 54 African nations

Country	Country code	ind.01 The percentage of people using at least basic drinking water services	ind.02 Percentage of people using safely managed sanitation services.	ind.03 Degree of IWRM implementation (1-100)	ind.04 Annual freshwater withdrawals, total (% of internal resources)	ind.05 Renewable internal freshwater resources per capita (cubic meters)	ind.06 Environmental flow requirements (106 m3/annum)	ind.07 Average precipitation in depth (mm per year)	ind.08 Access to electricity (% of population)	ind.09 Renewable energy consumption (% of total final energy consumption)	ind.10 Renewable electricity output (% of total electricity output)	ind.11 CO2 emissions (metric tons per capita)	ind.12 Electric power consumption (kWh per capita)	ind.13 Energy imports, net (% of energy use)	ind.14 Prevalence of undernourishment (%)	ind.15 Percentage of children under 5 years of age affected by wasting (%)	ind.16 Percentage of children under 5 years of age who are stunted (%)	ind.17 Prevalence of obesity in the adult population (18 years and older)	ind.18 Average protein supply (gr/caput/day)	ind.19 Cereal yield (kg per hectare)	ind.20 Average Dietary Energy Supply Adequacy (ADESA) (%)	ind.21 Average value of food production (I$ per caput)
Algeria	DZA	93.5	87.5	48.2	69.4	287.6	4.6	89.0	99.4	0.1	0.3	3.7	1,362.9	(177.1)	4.7	4.1	11.7	26.6	75.0	1,560.7	143.0	220.0
Angola	AGO	41.0	39.4	37.1	0.5	5,497.7	110.7	1,010.0	40.5	49.6	53.2	1.3	312.2	(541.0)	23.9	4.9	37.6	6.8	52.0	934.7	108.0	137.0
Benin	BEN	67.0	13.9	62.8	1.3	1,001.3	13.1	1,039.0	41.4	50.9	5.6	0.6	100.2	46.6	10.4	4.5	34.0	8.2	49.0	1,455.9	123.0	214.0
Botswana	BWA	79.2	60.0	41.1	8.1	1,106.7	2.7	416.0	60.7	28.9	0.03	3.2	1,815.6	44.5	28.5	7.2	31.4	16.1	64.0	452.8	98.0	172.0
Burkina Faso	BFA	53.9	22.5	62.6	6.5	710.8	3.0	748.0	19.2	74.2	9.4	0.2	n/a	n/a	21.3	7.6	27.3	4.5	61.0	1,181.4	122.0	122.0
Burundi	BDI	55.9	50.5	32.1	2.9	1,017.0	9.8	1,274.0	7.6	95.7	82.7	0.0	n/a	n/a	n.a.	5.1	55.9	4.4	n/a	1,519.6	n/a	109.0
Cabo Verde	CPV	86.5	65.2	64.3	6.8	569.9	n/a	228.0	92.6	26.6	20.2	0.9	n/a	n/a	12.3	n/a	n/a	10.6	69.0	178.0	113.0	73.0
Cameroon	CMR	65.3	38.8	33.8	0.4	12,275.2	213.4	1,604.0	60.1	76.5	76.1	0.3	275.2	(28.3)	7.3	5.2	31.7	9.5	56.0	1,643.7	126.0	244.0
Central African Republic	CAF	54.1	25.1	31.0	0.1	31,226.5	119.4	1,343.0	14.0	76.6	99.4	0.1	n/a	n/a	61.8	7.4	40.7	6.3	62.0	879.8	79.0	202.0
Chad	TCD	42.5	9.5	31.8	5.9	1,105.4	25.2	322.0	8.8	89.4	n/a	0.1	n/a	n/a	39.7	13.0	39.9	4.8	47.0	844.7	98.0	154.0
Comoros	COM	83.7	34.2	25.7	0.8	1,580.2	n/a	900.0	77.8	45.3	n/a	0.2	n/a	n/a	n.a.	11.1	32.1	6.9	n/a	1,355.8	105.0	90.0
Congo, Dem. Rep.	COD	41.8	19.7	31.3	0.1	12,207.9	981.7	1,543.0	17.1	95.8	99.8	0.1	108.5	2.0	n.a.	8.1	42.6	5.6	n/a	771.5	n/a	51.0
Congo, Rep.	COG	68.3	15.0	32.0	0.0	45,574.9	664.4	1,646.0	56.6	62.4	53.3	0.6	202.9	(496.6)	37.5	8.2	21.2	8.4	46.0	828.2	94.0	87.0
Côte d'Ivoire	CIV	73.1	29.9	32.1	2.0	3,410.4	61.3	1,348.0	64.3	64.5	16.7	0.5	274.7	7.1	20.7	6.0	21.6	9.0	58.0	2,133.9	119.0	271.0
Djibouti	DJI	76.9	51.4	n/a	6.3	328.9	n/a	220.0	51.8	15.4	n/a	0.8	n/a	n/a	19.7	21.5	33.5	12.2	59.0	1,925.6	108.0	78.0
Egypt, Arab Rep.	EGY	98.4	93.2	40.3	4,100.0	19.6	2.6	51.0	100.0	5.7	8.3	2.2	1,683.2	(7.4)	4.8	9.5	22.3	31.1	64.0	7,114.0	152.0	238.0
Equatorial Guinea	GNQ	49.6	74.5	24.2	0.1	23,020.6	15.2	2,156.0	67.9	7.8	57.8	4.7	n/a	n/a	n.a.	3.1	26.2	7.4	n/a	n/a	n/a	n/a
Eritrea	ERI	19.3	11.3		20.8	n/a	2.1	384.0	46.7	79.8	0.5	n/a	89.3	22.3	n.a.	15.3	50.3	4.1	n/a	414.2	n/a	47.0
Eswatini	SWZ	67.6	58.0	52.6	39.5	2,038.5	3.1	788.0	65.8	66.1	46.6	0.9	n/a	n/a	20.7	2.0	25.5	13.5	44.0	1,138.1	103.0	237.0
Ethiopia	ETH	39.1	7.1	31.3	6.4	1,253.0	89.3	848.0	42.9	92.2	100.0	0.1	69.2	5.9	21.4	9.9	38.4	3.6	26.0	2,484.0	105.0	114.0
Gabon	GAB	87.5	40.9	14.4	0.085	87,433.4	138.3	1,831.0	91.4	82.0	43.7	2.8	1,167.9	(213.4)	9.4	3.4	17.5	13.4	58.0	1,604.0	124.0	136.0
Gambia, The	GMB	80.1	41.7	29.8	3.0	1,564.3	3.4	836.0	47.8	51.5	n/a	0.3	n/a	n/a	9.6	11.1	25.0	8.7	72.0	840.7	120.0	68.0
Ghana	GHA	77.8	14.3	48.6	3.2	1,123.8	33.3	1,187.0	79.3	41.4	50.9	0.5	351.3	(8.2)	6.1	4.7	18.8	9.7	46.0	1,842.4	135.0	287.0
Guinea	GIN	67.4	22.0	24.1	0.2	19,143.6	161.0	1,651.0	33.5	76.3	78.8	0.2	n/a	n/a	19.7	8.1	32.4	6.6	61.0	1,180.0	115.0	174.0
Guinea-Bissau	GNB	69.2	21.5		1.1	9,271.4	19.7	1,577.0	14.7	86.9	n/a	0.2	n/a	n/a	26.0	6.0	27.6	8.2	63.0	1,426.4	102.0	213.0
Kenya	KEN	58.5	29.8	52.6	15.5	449.8	18.6	630.0	56.0	72.7	87.5	0.3	164.3	17.2	24.2	4.0	26.0	6.0	47.0	1,390.7	101.0	149.0
Lesotho	LSO	71.6	43.8	32.9	0.8	2,437.3	1.3	788.0	29.7	52.1	100.0	1.2	n/a	n/a	12.8	2.8	33.2	13.5	32.0	508.3	114.0	73.0
Liberia	LBR	69.9	16.9	15.0	0.1	45,550.4	176.8	2,391.0	19.8	83.8	n/a	0.2	n/a	n/a	38.8	5.6	32.1	8.6	60.0	1,322.3	101.0	74.0
Libya	LBY	96.8	99.7	46.9	822.9	112.8	n/a	56.0	98.5	2.0	n/a	9.2	1,811.1	(103.0)	n.a.	6.5	21.0	31.8	n/a	715.0	140.0	181.0
Madagascar	MDG	50.6	9.7	36.5	4.0	14,285.8	217.5	1,513.0	22.9	70.2	54.6	0.1	n/a	n/a	43.1	15.2	49.2	4.5	24.0	3,920.3	89.0	137.0
Malawi	MWI	67.2	43.5	40.3	8.4	945.6	9.5	1,181.0	11.0	83.6	91.3	0.1	n/a	n/a	26.3	2.7	37.1	4.7	39.0	1,347.4	104.0	139.0
Mali	MLI	74.3	31.3	53.3	8.6	3,537.1	55.2	282.0	35.1	61.5	43.5	0.1	n/a	n/a	6.0	13.5	30.4	7.1	62.0	1,607.5	142.0	244.0
Mauritania	MRT	69.6	44.6	45.4	337.0	98.4	1.2	92.0	41.7	32.2	13.4	0.7	n/a	n/a	11.3	14.8	27.9	11.3	78.0	1,221.6	126.0	153.0
Mauritius	MUS	99.9	93.1	64.4	26.4	2,181.7	n/a	2,041.0	98.8	11.5	22.7	3.4	2,182.5	84.5	5.8	n/a	n/a	11.5	92.0	3,455.0	125.0	190.0
Morocco	MAR	83.0	83.5	63.9	35.7	845.0	8.2	346.0	100.0	11.3	14.3	1.7	904.4	90.7	3.9	2.3	14.9	25.6	68.0	936.2	147.0	250.0
Mozambique	MOZ	47.3	23.6	54.6	0.9	3,685.8	133.0	1,032.0	24.2	86.4	86.4	0.3	478.9	(54.6)	30.5	6.1	43.1	6.0	41.0	823.8	106.0	97.0
Namibia	NAM	n/a	n/a	59.1	4.6	2,598.1	7.2	285.0	51.8	26.5	97.8	1.6	1,652.6	74.4	25.4	n/a	n/a	15.0	49.0	453.1	98.0	168.0
Niger	NER	45.8	12.9	49.7	28.1	182.8	10.6	151.0	16.2	78.9	0.8	0.1	51.2	(5.8)	14.4	10.3	42.2	4.7	55.0	530.3	123.0	180.0
Nigeria	NGA	67.3	32.6	35.1	5.6	1,252.4	157.2	1,150.0	59.3	86.6	18.2	0.5	144.5	(93.0)	11.5	10.8	43.6	7.8	57.0	1,443.6	117.0	211.0
Rwanda	RWA	56.7	62.3	34.7	1.6	837.3	10.3	1,212.0	29.4	86.7	56.9	0.1	n/a	n/a	36.1	2.2	37.9	4.8	26.0	1,522.5	100.0	209.0
São Tomé and Principe	STP	79.7	40.1	22.8	0.3	11,397.7	n/a	3,200.0	65.4	41.1	10.5	0.6	n/a	n/a	10.2	4.0	17.2	10.6	76.0	2,098.4	113.0	147.0
Senegal	SEN	75.2	48.4	53.3	8.6	1,773.7	20.2	686.0	64.5	42.7	10.4	0.6	229.4	52.7	11.3	7.2	17.0	7.4	72.0	1,349.0	111.0	103.0
Seychelles	SYC	96.3	100.0	45.0	n/a	n/a	n/a	2,330.0	100.0	1.4	2.4	5.4	n/a	n/a	n.a.	4.3	7.9	14.6	n/a	n/a	99.0	53.0

Sierra Leone	SLE	58.1	14.5	18.6	0.1	22,601.5	117.2	2,526.0	20.3	77.7	61.0	0.2	n/a	n/a	25.5	9.4	37.9	7.5	57.0	1,889.1	109.0	177.0
Somalia	SOM	40.0	16.2	10.3	55.0	444.0	1.3	282.0	29.9	94.3	n/a	0.0	n/a	n/a	n.a.	15.0	25.3	6.9	n/a	507.3	87.0	123.0
South Africa	ZAF	84.7	73.1	65.5	34.6	821.4	20.1	495.0	84.2	17.2	2.3	9.0	4,197.9	(14.5)	6.1	2.5	27.4	27.0	83.0	3,809.5	123.0	229.0
South Sudan	SSD	50.4	10.4	38.3	2.5	2,254.8	33.9	900.0	8.9	39.1	0.6	0.1	43.6	(1,058.1)	n.a.	22.7	31.1	n/a	n/a	1,511.8	n/a	146.0
Sudan	SDN	58.9	34.6	39.9	673.3	101.7	15.1	250.0	38.5	61.6	64.5	0.3	190.2	(9.0)	25.2	16.3	38.2	7.4	69.0	684.8	106.0	163.0
Tanzania	TZA	50.1	23.5	50.3	6.2	1,608.1	56.3	1,071.0	32.8	85.7	34.2	0.2	103.7	10.7	32.0	4.5	34.4	4.1	46.0	1,540.7	106.0	193.0
Togo	TGO	62.8	13.9	31.9	1.5	1,590.8	8.1	1,168.0	46.9	71.3	75.3	0.4	154.7	20.0	16.2	6.7	27.5	7.1	49.0	1,131.4	114.0	122.0
Tunisia	TUN	94.2	93.1	54.5	76.7	376.4	0.7	207.0	100.0	12.6	2.8	2.6	1,454.6	36.2	4.9	2.8	10.1	27.3	90.0	1,541.7	142.0	358.0
Uganda	UGA	38.9	19.2	58.7	1.6	1,004.3	49.2	1,180.0	26.7	89.1	93.0	0.1	n/a	n/a	41.4	3.6	28.9	7.1	47.0	1,906.2	95.0	120.0
Zambia	ZMB	61.2	31.1	46.1	2.0	5,134.1	49.4	1,020.0	27.2	88.0	97.0	0.3	717.3	8.3	44.5	6.3	40.0	6.5	41.0	2,418.0	93.0	118.0
Zimbabwe	ZWE	66.6	38.6	61.0	29.1	795.5	9.3	657.0	38.1	81.8	52.7	0.8	609.1	15.3	46.6	3.2	26.8	12.3	58.0	580.0	87.0	75.0
